# Long-term Surgical Outcome of Congenital Pseudarthrosis of the Tibia

**DOI:** 10.5704/MOJ.2507.010

**Published:** 2025-07

**Authors:** TJ Ong, K Jamil, AF Abd-Rasid, AH Abdul-Rashid, S Ibrahim

**Affiliations:** Department of Orthopaedics and Traumatology, Universiti Kebangsaan Malaysia, Kuala Lumpur, Malaysia

**Keywords:** congenital pseudarthrosis tibia, tibial pseudoarthrosis, intramedullary rodding, Ilizarov external fixator

## Abstract

**Introduction:**

Congenital pseudarthrosis of the tibia (CPT) is challenging to treat. The main issues following surgery are non-union, refracture, limb deformity and length discrepancy. We evaluated the surgical outcome of children operated in our centre.

**Materials and methods:**

A retrospective study of the outcome of primary bone union, refracture and success rate. Patients who had reached skeletal maturity were further evaluated for Johnston grading, residual limb deformity and limb length discrepancy (LLD).

**Results:**

Twelve patients (13 tibiae) were reviewed with an average follow-up of 14.5 years (range 3.1-24.0 years). Nine (69.2%) tibiae underwent intramedullary (IM) rodding; two (15.4%) were stabilised with the Ilizarov external fixator (IEF) + IM rod; and two other (15.4%) tibiae with the IEF only. Primary union was achieved in 5 (38.5%) tibiae, but refractures occurred in two tibiae (40%), lowering the overall success rate to 23.1%. Fixation with IM rodding alone led to a low primary union rate (22.2%) but combining it with IEF avoided refracture. Seven (53.8%) tibiae reached skeletal maturity and had a union at 12.6 years (7.5–17.4 years), after an average of 3 surgical procedures. Four (57.1%) were Johnston Grade I, and 3 (42.9%) were Grade II. Four (57.1%) tibiae had residual tibial valgus, two (28.6%) tibial varus, four (57.1%) procurvatum and one (14.3%) recurvatum. The average LLD was 3.9cm (2-10cm).

**Conclusion:**

Intramedullary rodding alone is ineffective for producing a bony union but combining it with IEF minimise the refracture rate. The chances of union increased with age, but residual deformity and shortening are an ongoing challenge.

## Introduction

Congenital pseudarthrosis of the tibia (CPT) is a rare dysplastic pathological disorder of the lower limbs that causes substantial disability. Approximately 65% of CPT children had NF-11. The natural course of CPT is quite unfavourable. The affected area consists of fibrous hamartoma with high osteoclastogenicity and low osteogenecity properties^2^. There is little to no propensity for the lesion to heal on its own once a fracture has occurred. The key to getting primary union is to excise hamartomatous tissue and pathological periosteum. Age at surgery, status of the fibula, associated shortening, and deformities of the leg and ankle play significant roles in primary union and residual challenges after primary healing^3,4^.

Following the excision of hamartomatous tissue, CPT is commonly stabilised using one of four surgical techniques: intramedullary (IM) rodding, Ilizarov external fixator (IEF), combination IEF and IM rodding or vascularised fibular graft. Although IEF and IM rodding combined have shown a lower refracture rate than IEF alone, the overall success rate is still not encouraging^5^. The overall success probability for attaining bone union without refracture using these four procedures is approximately 50%^1,6,7^. In our centre, the choice of primary surgery is IM rodding or combining with an IEF after resection of the dysplastic segment.

The purpose of this study is to evaluate the union rate, refracture rate, and residual deformity following surgery as several children treated in our centre have reached skeletal maturity.

## Materials and Methods

**Fig. 1:**
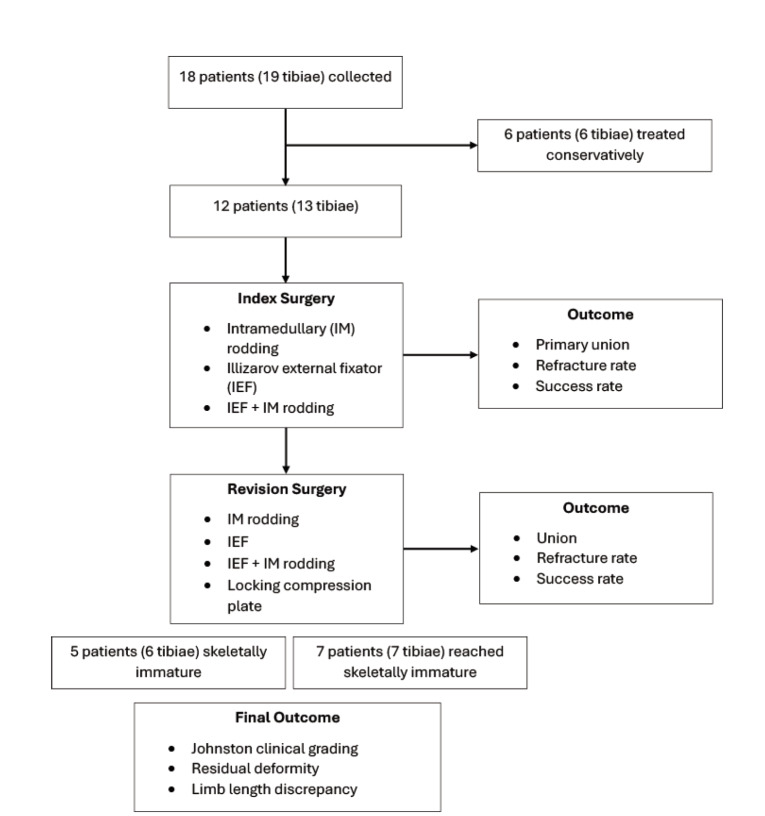
Flowchart of the patient selection and study design.

A retrospective study of congenital pseudarthrosis of the tibia (CPT) patients who received care at our centre between 1996 and 2019 was carried out. The inclusion criteria were all CPT patients who underwent surgical treatment. This study was approved by the Ethical Committee, Faculty of Medicine, Universiti Kebangsaan Malaysia (JEP-2021-376). This study was designed to evaluate patient outcomes following index surgery in terms of primary union rate, refracture rate, and success rate. Further investigations were done to study the long-term surgical outcomes on both the index and revision surgeries, residual deformity, limb length discrepancy (LLD), and Johnston grading at the time of skeletal maturity. Radiographs and the patient’s case notes were examined. Gender, ethnicity, age at diagnosis, age at first operation, and whether the children had reached skeletal maturity were all recorded as demographic information. The closure of the epiphyseal plate on tibial radiographs was used to determine skeletal maturity. Fig. 1 shows the flowchart of the study design.

The tibial radiographs of the patients were reviewed and categorised using Crawford’s classification system, which identified four types of CPT, all of which feature anterolateral tibial bowing^8^. A diagnosis of neurofibromatosis type 1 (NF-1) was made with National Institutes of Health (NIH) clinical criteria^9^. The presence of union, refracture, residual deformity, and the presence of limb length discrepancy were examined in follow-up clinical notes and radiographs.

In the majority of cases, the choice of index surgery at our centre was IM rod insertion and casting after resection of the fibrous hamartoma. We performed trans-ankle intramedullary fixation and acute apposition following the methods of Charnley and Williams^10,11^. The intramedullary implant used to stabilise the apposition site was either a Steinmann pin, Rush rod, or K-wire.

When there was inadequate bone contact area, particularly with the severe atrophic type of CPT, a bone graft was used. Our options for the bone graft were the fibula, the proximal tibia of the ipsilateral limb, and the iliac crest. IEF together with/without an intramedullary rod, was used to treat a large bone defect. Post-operatively, a long leg cast was applied for four to six weeks followed by a patella tendon-bearing cast. Once the bone had consolidated and the angular deformity corrected, a protective orthosis was applied. For subsequent surgeries, various methods were employed, including a locked plate.

The bone union was determined by the radiological presence of bridging callus at 3 out of 4 cortices on anteroposterior and lateral views^12^. The Johnston clinical grade system (Table I), a clinical and radiographic outcome assessment, was used to evaluate the CPT's endpoint at the latest follow-up visit^3^. The limb length discrepancy data was obtained from the case notes, while the radiographs were used to measure the angular deformity of the tibia.

**Table I:** Johnston clinical grading system for congenital pseuarthrosis of the tibia^13^.

Grade 1	• Unequivocal union
	• Full weight bearing function
	• Maintenance of alignment requiring no additional surgical treatment
Grade 2	• Equivocal union (residual transverse or longitudinal cortical deficiency) with useful function
	• Limb protected by a brace
	• >15° valgus, tibial procurvatum or recurvatum for which additional surgery was required or anticipated
Grade 3	• Persistent non-union or refracture
	• Requiring full time external support for pain or instability

The anteroposterior and lateral views of the tibial radiographs were used to measure the amount of residual angular deformity following bone union. The difference between the angles of the medial proximal tibial angle (MPTA) and the lateral distal tibial angle (LDTA) was used to calculate the tibial coronal plane deformity. For sagittal plane deformity, posterior proximal tibial angle (PPTA) was assessed. Tibial procurvatum is defined as a posterior tibial slope of more than 13°, whereas tibial recurvatum is defined as an anterior tibial slope of more than 6°^13^.

## Results

A total of 18 patients had a reported diagnosis of CPT during the study period. Six patients who did not undergo surgical treatment at our centre were excluded from the study. The remaining 12 patients (13 tibiae) made up the study group. The mean age at diagnosis was 1.1±0.83 years, and mean age at first surgery was 3.2±1.83 years. There were 7 (58.3%) boys and 5 (41.7%) girls. Eleven patients had unilateral CPT (6 right, 5 left) and one bilateral. Nine patients had NF Type 1, while the other 3 did not. The mean follow-up duration was 14.5 years (range of 3.1 to 24 years). At the most recent follow-up, seven (58%) children had reached skeletal maturity.

Almost half of the tibiae, (46.1%) were Crawford Type II-C (atrophic type) associated with fibular pseudarthrosis. Two (15.4%) and five (38.5%) tibiae, respectively, were Crawford Type II-B and Type II-A. There was fibular pseudoarthrosis in 7 (53.8%) cases. All 13 (100%) CPT sites were located at the distal third of the tibia shaft.

Nine (69.2%) tibiae received IM rodding as the index surgery, two (15.4%) underwent IEF and two (15.4%) had IEF in combination with an IM rod. In 5 out of 13 tibiae (38.5%), the primary union took place after an average of 9.2 months. The primary union rate for the IM rodding technique alone was low (22.2%) or 2 out of 9 tibiae. Refractures happened in 2 of the 5 tibiae after an average of 8.9 years (7.0-10.7 years), for a refracture rate of 40%. Combining IM rod and the IEF avoided refractures. The overall success rate for bone union without refractures was 23.1 %, or 3 out of 13 tibiae. The outcome of the different type of procedures are listed in Table II.

**Table II:** Index surgery, union, refracture and success rate for the 12 patients (13 tibiae).

Surgery	Number of cases		Mean age surgery (years)	Primary union		Refracture		Probability success rate
	N	%		N	%	N	%	%
IM rodding	9	69.2	2.4	2	22.2	1	50.0	11.1
IM rod with IEF	2	15.4	5.0	1	50.0	0	0	50.0
IEF alone	2	15.4	4.0	2	100.0	1	50.0	50.0
Total/Mean	13	100.0	3.2	5	38.5	2	40.0	23.1

Abbreviations – IM: intramedullary, IEF: Ilizarov external fixator

**Fig. 2:**
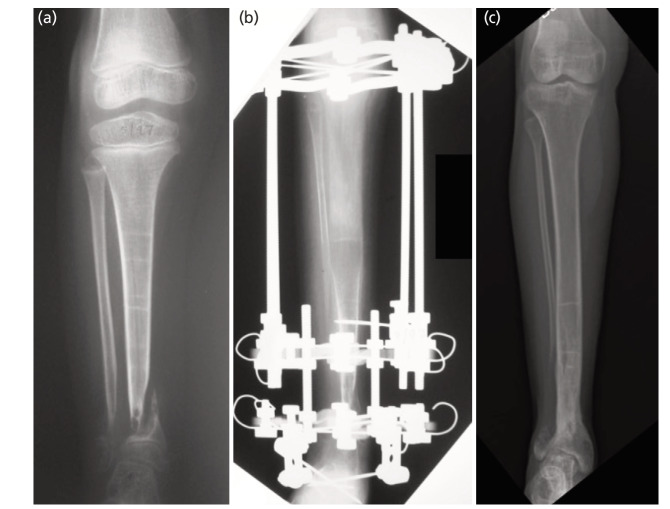
Tibial radiographs of Case 1. Bone union was obtained at age 7 and no refractures. She had mild residual deformity - Johnston Grade I: (a) Congenital pseudoarthrosis of the tibia and fibula at age 6 years. (b) IEF lengthening at age 6 years. (c) Distal tibial valgus and 2cm LLD at age 23 years.

**Fig. 3:**
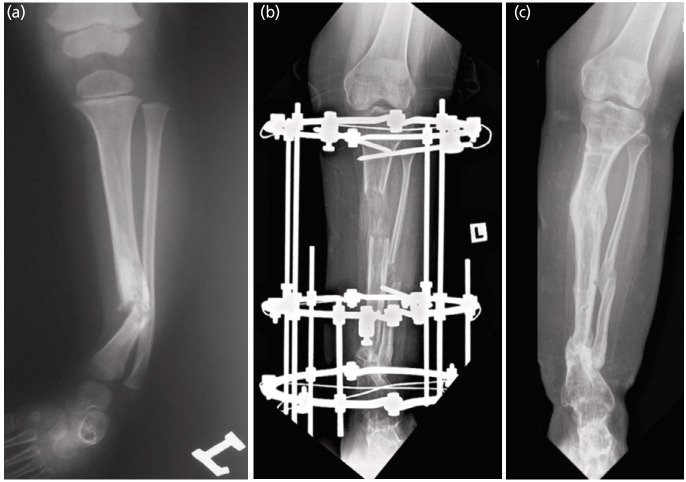
Tibial radiographs of Case 7. She had four surgical procedures since the age of 2 years, refractured twice and achieved union at age 17 years. (a) Congenital pseudoarthrosis of the tibia at age one year. (b) IEF lengthening at age 17 years. (c) Age 19 years with 4cm LLD and 24o distal tibial valgus – Johnston Grade II.

A total of 22 IM rodding procedures were performed to treat our patients. Nine out of 22 (40.9%) IM rodding procedures using autologous bone grafts had a combined union rate of 33.3%. The graft was harvested from four (44.5%) iliac bones, three (33.3%) proximal tibias, and two (22.2%) fibular bones. Union was achieved in 2 out of 4 cases for iliac bone graft (50%), 1 in 3 cases for tibia (33.3%), and none for fibula autograft. Interestingly, thirteen other IM rodding procedures without bone graft was associated with a higher union rate of 38.4%. At the time of the study, seven patients (58.3%) had reached skeletal maturity (case 1-7). They had a total of 21 operations: 13 (61.9%) IM rodding, 4 (19%) IM rodding + IEF, 3 (14.3%) IEF alone, and 1 (4.8%) locking compression plate—were carried out to establish union (Table III). For these patients, union was achieved at a mean age of 12.6 years (range 7.5-17.4 years) and an average of 3 operations (1–5 years). There was an average residual limb length discrepancy of 3.9cm (2-10cm). Four (57.1%) tibiae had residual tibial valgus with a mean angle of 19.6° (11.4-30.7°), while two (28.6%) tibiae had tibial varus with a mean of 9.2° (range: 7.3-11.0°); four (57.1%) had tibial procurvatum with a mean of 18.0^0^(range: 14.0-21.1°), while one (14.3%) had recurvatum of 6.5°. At the endpoint evaluation, four (57.1%) were Johnston Grade I and the remaining three (42.9%) were Johnston Grade II. Table IV summarises the clinical data for all patients. Fig. 2 and 3 are radiographs of two patients who had reached skeletal maturity.

**Table III:** Index and revision surgery to obtain union in seven skeletally matured patients.

Surgery goal to achieve union	Number of cases		Union		Refracture		Probability success rate
	N	%	N	%	N	%	%
Intramedullary rodding	13	61.9	6	46.2	2	33.3	30.8
IM rod with IEF	4	19.0	2	50.0	0	0	50.0
IEF alone	3	14.3	3	100.0	1	33.3	66.7
Locking compression plate	1	4.8	1	100.0	0	0	100.0
Total	21	100.0	12	57.1	3	25.0	42.8

Abbreviations – IEF: Ilizarov external fixator, IM: intramedullary

**Table IV:** Clinical data and the final outcome for all patients (13 tibiae).

Case	Gender	Crawford type	Side	NF1	Fibular pseudo-arthrosis	Age at Surgery (yr + mo)	Index Surgery	Number of surgeries for bone union	Age CPT union (yr + mo)	Years of follow- up (Age at last follow up: yr + mo)	Outcome: Johnston clinical evaluation	Residual LLD and deformity
1	F	II-C	R	No	Yes	6 + 7	Ilizarov proximal tibial epiphyseal distraction	0	7 + 6	24 (25 + 4)	Grade I	Procurvatum 6.8°
2	M	II-C	R	Yes	Yes	1 +4	Ilizarov external fixation	1	17 + 5	17.5(18 + 9)	Grade I	Recurvatum 5.3°
3	M	II-C	R	Yes	Yes	4 + 1	Ilizarov proximal tibial lengthening, compression of CPT site & IM K-wire	2	11	18 (18)	Grade II	Procurvatum 18.3°.
4	F	II-A	R	Yes	No	2+4	IM K-wire and fibular osteotomy	3	13	21.8 (21 + 10)	Grade I	LLD: 2cm. Varus 7.3°. Recurvatum 6.5°
5	M	II-A	R	Yes	No	4+10	Intramedullary Steinmann pin tibia and k-wire fibula, osteotomy fibula	3	13	17.3 (19 + 11)	Grade II	LLD: 3cm. Varus 11°. Procurvatum 21.1°
6	M	II-C	L	Yes	Yes	1 +4	Intramedullary K-wire tibia and fibula	1	9+ 1	16.8 (22+5)	Grade I	LLD: 3cm. Valgus 12°. Procurvatum 14°
7	F	II-B	L	No	No	1 + 10	IM Steinmann pin, double onlay fibular graft and corrective osteotomy (middle segment inverted)	4	17 + 3	18.4 (19 + 7)	Grade II	LLD: 4cm. Valgus 24.4°. Procurvatum 18.7°
8	M	II-A	L	Yes	No	2+7	Intramedullary Steinmann pin and onlay fibular graft	4	Non-union	13.4 (15 + 10)	Grade III	LLD: 15cm
9	M	II-C	L	No	Yes	6	Ilizarov proximal tibial lengthening, compression of CPT site and IM Steinmann pin tibia, K-wire fibula	0	6 + 11	9.8 (13 + 9)	Grade I	LLD: 7cm. Valgus 14°
10	F	II-A	L	Yes	Yes	1 + 5	IM Steinmann pin and corrective osteotomy (middle segment inverted)	2	Non-union	9.5(11)	Grade III	LLD: 2cm. Valgus 30°
11A	M	II-C	R	Yes	Yes	2+7	Intramedullary Steinmann	2	4 + 1	4.8 (6 + 8)	Grade II	No LLD
11B		II-B	L		No	2+7	Intramedullary Steinmann pin and fibular osteotomy	1	3 + 10	4.8 (6 + 8)	Grade II	Recurvatum 20°
12	F	II-A	R	Yes	No	4 + 1	Intramedullary Steinmann pin and fibular osteotomy	0	3 + 3	3.1 (4+1)	Grade II	Valgus 12°

## Discussion

The management of CPT remains a challenging problem in children. The pseudarthrosis appears to be resistant to union, especially in the younger age group^14^. The four popular operative techniques: intramedullary (IM) rodding, Ilizarov external fixation (IEF), IM rodding combined with IEF or vascularised fibular graft, have varying success^15^, and refractures are common.

The choice of index surgery at our centre was IM rod insertion and casting following pseudarthrosis excision. Variations in IM rodding technique resulted in inconsistent results across studies. According to Paley *et al*, the average primary union rate of IM rodding surgery was 61%, ranging from 21% to 95% from different published studies; whilst the average refracture rate was 24%; with an overall success rate of 40%^5^. The benefit of IM rodding is that once the bone heals, the intramedullary rod acts as an internal splint to keep the bone from refracturing. By comparison, the average refracture rate for the IEF technique alone is much higher, at 41%^5^. In our series of IM rodding index surgeries, only 2 out of 9, or 22.2%, managed to achieve primary union at a mean of 11 months following surgery. Refracture occurred in one of the two primary bone unions (Case 6), yielding a low success rate of 11.1%. The refracture was managed with revision to a bigger IM rod and additional bone graft to achieve union. Despite this low union rate, in most of the children stabilisation of the tibia allowed ambulation with the support of an ankle-foot orthosis.

Singer *et al* stressed the significance of fibular surgery in a technique of IM rodding, which can greatly improve surgical success based on Johnston grading, which assesses union, angular deformity, and weight-bearing status^16^. In comparison to the groups of patients who did not undergo fibular resection or osteotomy procedures, the long-term prognosis is noticeably better in the groups of patients who underwent fibular shortening procedures with or without fixing the fibula with an intramedullary K-wire^16^. The tibia is frequently kept distracted by an intact fibula, and fixation is difficult when there is a fibular deformity or a big segment loss due to fibular pseudarthrosis. To allow proper docking of the pseudoarthrosis site, an intact fibula is osteotomized or resected. The healthy fibula can be used as an onlay graft and placed at the site of the pseudoarthrosis. Three of our patients had intramedullary fibular K-wires performed in conjunction with the index surgery. Following fibular osteotomy, 5 out of 8 (62.5%) fibular united.

Bone grafting was not routinely done for our patients. Most were treated with acute docking of the resected bone ends. However, the IM rodding union rate with bone grafting was low, at 33.3%, compared to without bone graft (38.4%). As such, we are unable to conclude whether bone grafting was helpful in our cases. Other authors had different results. Dobbs *et al* concluded that IM rodding with bone graft had a primary union rate of 86% and a refracture rate of 57% out of 21 tibiae with a mean follow-up of 14.2 years^17^. The size of the bone gap following index surgery would influence the treatment strategy. A previous recommendation was that without a bone gap, the CPT site could be treated with bone grafting, while cases with a bone gap would be treated by bone transport, bone lengthening, or a vascularised fibular graft^18^. Kesireddy *et al* found that a combined technique using cortical autograft had a statistically significant reduction in the number of refractures in comparison to stand-alone fixation methods such as IM rodding and IEF^1^.

We had two patients who were treated with a proximal tibial corticotomy and IEF combined with an IM rod (Case 3 and Case 9). Augmenting IM fixation with an external fixator can provide compression at the tibial pseudarthrosis site^19-21^. The aim of this combined method is to benefit from the rod’s protective effect and the IEF device’s high rate of union and alignment control^19,22^. One of the two patients obtained primary bone union without a refracture until skeletal maturity (Case 9). In Cases 3 and 4, a repeat lengthening procedure with IEF without compression at a later age, led to bony union. We reported these findings previously^23^, and both cases had no refracture and have reached skeletal maturity. Meanwhile, IEF alone was performed in two cases (Case 1 and 2). Case 1 had primary union and no refractures, with a good outcome at skeletal maturity. However, Case 2 had refracture after achieving primary union and was treated with a locking plate in combination with a synthetic bone graft and reverse sural flap at a later age. This case was also reported previously^24^.

Another prognostic factor affecting the outcome for CPT is the patient’s age^14,25^. Skeletal maturation is thought to provide some protection to the tibia^25^. Therefore, the final outcome of CPT should be assessed after skeletal maturity^17^. In our series, we have 7 skeletally mature patients at the time of the last follow-up; all achieved union at an average age of 12.6 years, with various surgical methods utilised. In a multicentre study in Europe, the European Paediatric Orthopaedic Society (EPOS) recommended avoiding surgery for CPT in patients younger than three years old, or if possible, the operation should be delayed to the age of five years^14^. A long-term study of 12 patients treated with IEF bone transport revealed that the best results were achieved in those operated between the ages of 4 to 10 years^26^. In contrast, another study from China found significantly better results in children operated below three years of age, as compared to older children^27^. We believe that the index surgery to stabilise the limb can be done at an early age. However, if the non-union persists, the subsequent surgery can be performed when the limb has significant shortening, which is usually at an age nearer to skeletal maturity.

The type and location of CPT are factors that can influence the outcome. Failure of primary union in our cases might be due to a distally located atrophic lesion. After resection of the fibrous hamartoma, stable fixation was difficult because of the small bone stock remaining distally and the large tibial bone gaps. Stability is largely determined by the IM rod through the ankle and subtalar joints. Kim *et al's* retrospective study on 12 CPTs encountered a similar condition: all the distally located CPTs failed to achieve primary union^28^.

Half of our CPT (46.1%) was Crawford type II-C which have the worst functional results. Other studies reported this CPT type led to severe leg length discrepancies, needed permanent bracing, and had ankle joint functions that were fair or poor^29^.

Due to generally unsatisfactory results across all surgical techniques, adjunct treatments have been widely investigated. These included antiresorptive and osteoinductive agents. It was proposed that pharmacological treatment of CPT with bisphosphonates must be combined with an additional osteoinductive stimulus to enhance bone formation. The mesenchymal stem cells in the CPT patients were found to have less differentiation potential and less osteogenic potential than normal bone marrow stem cells^30^. When combined with an antiresorptive drug, bone morphogenic proteins (BMP) may have a synergistic effect in the treatment of CPT^31,32^. Most recently, Paley’s cross union surgical protocol with success rates of up to 100% are promising. Pharmacological treatment with antiresorptive agents and BMP in the Paley cross-union treatment protocol is aimed at upregulating the sluggish CPT bone osteogenesis^7^. We have no experience with this technique.

This study is limited by the retrospective design with a small sample and non-standardised surgical methods which may have influenced the outcomes. Nevertheless, it adds to the literature the outcome of surgical treatment for CPT at skeletal maturity.

## Conclusion

Our results suggested that the index surgery with IM rodding method alone is ineffective for obtaining primary unions compared with other methods (IEF ± rodding). However, the internal splinting effect from intramedullary rodding plays a role in reducing the chances of refractures, especially when combined with the IEF. In the long-term, bone union does occur with a reasonably good outcome (Johnston Grade I or II).
